# Modulation of the Neuregulin 1/ErbB system after skeletal muscle denervation and reinnervation

**DOI:** 10.1038/s41598-018-23454-8

**Published:** 2018-03-22

**Authors:** Michela Morano, Giulia Ronchi, Valentina Nicolò, Benedetta Elena Fornasari, Alessandro Crosio, Isabelle Perroteau, Stefano Geuna, Giovanna Gambarotta, Stefania Raimondo

**Affiliations:** 1 Department of Clinical and Biological Sciences, University of Torino, 10043 Orbassano, Italy; 2 Neuroscience Institute Cavalieri Ottolenghi (NICO), University of Torino, 10043 Orbassano, Italy; 3 Microsurgery Unit, AOU Città della Salute e della Scienza, PO CTO, 10126 Torino, Italy

## Abstract

Neuregulin 1 (NRG1) is a growth factor produced by both peripheral nerves and skeletal muscle. In muscle, it regulates neuromuscular junction gene expression, acetylcholine receptor number, muscle homeostasis and satellite cell survival. NRG1 signalling is mediated by the tyrosine kinase receptors ErbB3 and ErbB4 and their co-receptors ErbB1 and ErbB2. The NRG1/ErbB system is well studied in nerve tissue after injury, but little is known about this system in skeletal muscle after denervation/reinnervation processes. Here, we performed a detailed time-course expression analysis of several NRG1 isoforms and ErbB receptors in the rat *superficial digitorum flexor* muscle after three types of median nerve injuries of different severities. We found that ErbB receptor expression was correlated with the innervated state of the muscle, with upregulation of ErbB2 clearly associated with the denervation state. Interestingly, the NRG1 isoforms were differently regulated depending on the nerve injury type, leading to the hypothesis that both the NRG1α and NRG1β isoforms play a key role in the muscle reaction to injury. Indeed, *in vitro* experiments with C2C12 atrophic myotubes revealed that both NRG1α and NRG1β treatment influences the best-known atrophic pathways, suggesting that NRG1 might play an anti-atrophic role.

## Introduction

The innervation of skeletal muscle has a predominant role in muscle structural and functional maintenance. Therefore, peripheral nerve damage leads to wide molecular and structural modifications of target muscle fibres. Denervated myofibres undergo atrophy, a condition of loss of balance between protein synthesis and protein degradation, with consequent loss of muscle strength, mass and myofibre diameter, and activation of the apoptosis program and autophagy-lysosome machinery^1,2^. Atrophy is caused by an imbalance of protein production and protein degradation, due to the activation of genes known as “atrogenes”, such as atrogin and muscle RING finger 1 (murf1), which encode two E3 ubiquitin ligases responsible of the degradation of several proteins^3,4^. Moreover, the stem cell pool in the muscle, the satellite cells, exit from their quiescent state and become active: they proliferate and then differentiate into multi-nucleated myofibres. This mechanism tries to maintain the muscle mass until reinnervation. When reinnervation occurs, all the features of atrophic state completely or partially recede. Thus, after a traumatic nerve injury of small entity, the immediate nerve repair usually induces a complete recovery of nerve tissue with good muscle functional recovery. However, after a traumatic nerve injury of great entity or when nerve repair is delayed for surgical reasons, post-denervation changes in muscle tissue aggravate the patient’s clinical scenario^5^. The progressive decline of denervated muscle is associated with the loss of satellite cells and Schwann cells, followed by reduced growth factor release and reduced ability to guide regenerated axons to the correct fibres. Moreover, the tissue is progressively substituted by fat and fibrotic tissue^5^.

Neuregulin 1 (NRG1) is a trophic factor produced by nerves as well as by the muscle. It is a member of the epidermal growth factor (EGF) family, and it mediates the crosstalk between motor axon and muscle, between terminal Schwann cells and motor axon, and between different muscle fibres^6^. Alternative splicing gives rise to several NRG1 isoforms, classified as soluble (types I, II) or transmembrane (type III) isoforms. A more detailed classification divides NRG1 in α or β isoforms on the basis of a small domain proximal to the EGF-like domain, or into type a, b, and c isoforms depending on the exon included at the C-terminal tail^7^. NRG1 signalling is mediated by the ErbB tyrosine kinase receptors ErbB3 and ErbB4 and their co-receptors ErbB1 and ErbB2. All the receptors are mainly present at the neuromuscular junction^8^.

NRG1 regulates myogenesis^9^, muscle spindle development and maintenance^10^, glucose transport^11,12^, mitochondrial oxidative capacity^13,14^ and acetylcholine receptor expression^8,15^. A role of the NRG1/ErbB system in muscle regeneration has been postulated, and NRG1 expression increases in satellite cells and motoneurons after toxin-induced muscle damage^16^. The role of the NRG1/ErbB system during muscle denervation and reinnervation after nerve injury is far from being understood, since only incomplete and discordant data are available. Here, we performed a detailed time-course expression analysis of different NRG1 isoforms and ErbB receptors in the rat *superficial digitorum flexor* (SDF) muscle after median nerve injury, comparing three types of injury. Moreover, to investigate the effects of NRG1α and NRG1β, *in vitro* stimulation experiments were performed using C2C12 cells as a model of atrophic myotubes.

## Results

### Short-term analysis of muscle after denervation and reinnervation

#### Morphological and morphometrical analysis

**Figure 1:**
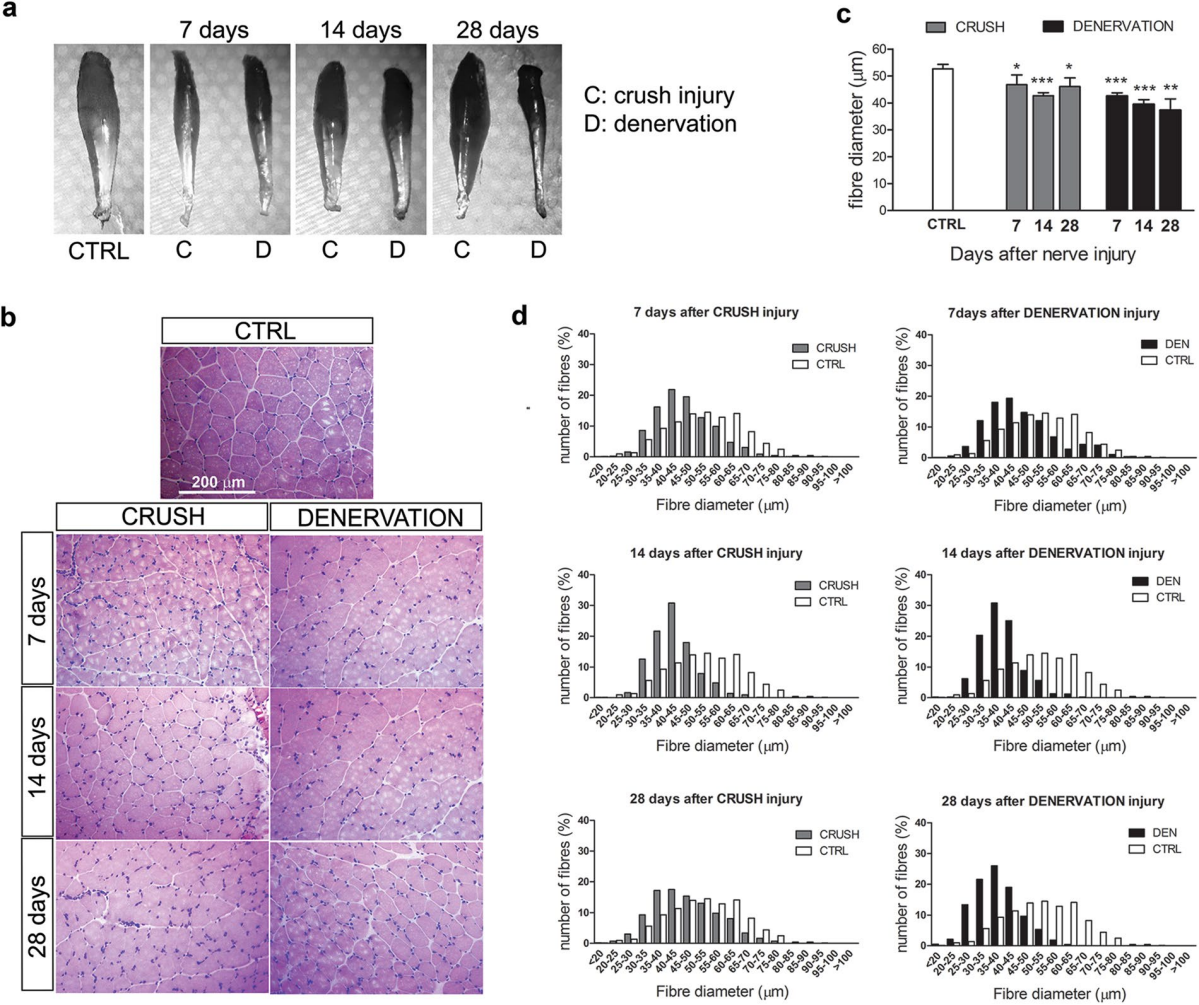
Morphometrical analysis of SDF muscles after nerve injury. (**a**) Representative SDF muscles as they appear after nerve injury. (**b**) Representative transverse sections of muscle, stained with haematoxylin-eosin, in the control condition and 7, 14 and 28 days after median nerve injury (crush injury or denervation). (**c**) Graphs representing results of morphometrical analysis performed on muscle sections. The mean fibre diameter is reported for each group at different time points after nerve injury. The analysis was performed on four animals per group; data are expressed as the mean ± SD. Statistical analysis: Student’s T-test: each value versus control muscle. *p ≤ 0.05; **p ≤ 0.01; ***p ≤ 0.001. (**d**) The graphs show the muscle fibre size distribution in injured muscle (grey: crush nerve injury; black: denervation) together with the distribution observed in un-injured muscle (white); un-injured n = 732; denervation 7 days n = 855, 14 days n = 838, 28 days n = 973; crush 7 days n = 882, 14 days n = 724, 28 days n = 701.

A morphological analysis of the SDF muscle was performed after median nerve injury. Macroscopic analysis revealed muscle atrophy in denervated muscle with loss of muscle mass from 7 to 28 days, while in the crush group the mass loss was rescued at 28 days (Fig. 1a). Morphometric analysis shows that the mean fibre diameter was reduced with respect to un-injured muscle for all the investigated time points in both crush and denervated groups; however, in the denervated group fibre diameter decreased over time, while in the crushed group it stopped decreasing at 28 days (Fig. 1b,c). Indeed, 28 days after nerve injury the fibre size distribution was similar between the crush group and the un-injured muscle, while in the denervated group the histograms were shifted to the left with a higher number of small fibres (Fig. 1d).

#### Regulation of the NRG1/ErbB system

**Figure 2:**
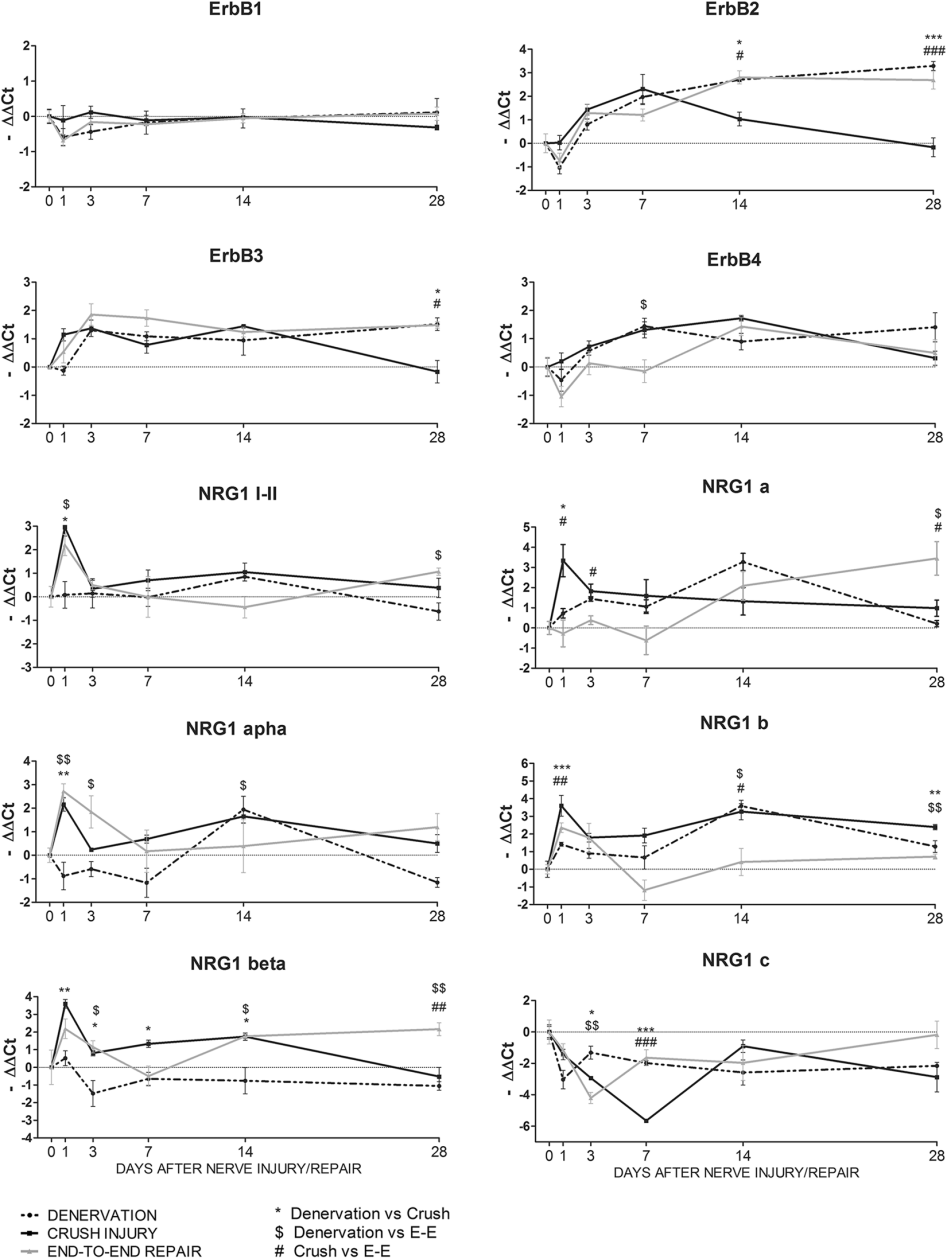
The expression of ErbB receptors and NRG1 isoforms is modulated in muscle after nerve injury. Three different types of nerve injury and injury/repair were performed on the median nerve (denervation, crush and end-to-end repair). At different time points, SDF muscle was collected and used for expression analysis. Graphs show the results of quantitative real-time PCR analysis. The analysis was performed on three animals per group; data are expressed as the mean ± SEM. Statistical analysis comparing groups at each single time point: one-way ANOVA plus Bonferroni’s post hoc test: *p ≤ 0.05; **p ≤ 0.01; ***p ≤ 0.001. E-E: end-to-end repair.

A time-course mRNA expression analysis of the NRG1 isoforms, ErbB3 and ErbB4 receptors, and their co-receptors ErbB2 and ErbB1, was performed on SDF muscle after median nerve injury (Fig. 2).

The results show that ErbB1 mRNA expression was not regulated after the muscle denervation or denervation/reinnervation process. In contrast, ErbB2 mRNA expression changed significantly in the three experimental models investigated. In the denervated and end-to-end repair groups, ErbB2 mRNA was upregulated compared to un-injured muscle from day 7 or 14 until day 28 (see Table S1 for statistical analysis). In the crush group, ErbB2 mRNA showed an early upregulation, with a peak 7 days after injury and a returning to baseline around day 14. Statistical analysis revealed significant differences between groups in the later phase. ErbB3 expression was similar in the denervated and end-to-end repair groups: in the denervated group ErbB3 mRNA was upregulated 3, 7 and 28 days after injury, as well as in the end-to-end repair group, where the upregulation was detectable at days 3 and 28. In the crush group, ErbB3 mRNA expression significantly increased 3 and 14 days after injury and returned to baseline at 28 days, when the differences between groups were appreciable. Finally, ErbB4 mRNA expression did not change significantly in the denervated or in the end-to-end repair group, whereas in the crush group it was upregulated 7 and 14 days after injury, followed by a return to an expression level comparable to the control.

The expression of the soluble isoforms of NRG1 (NRG1 type I and II) was also assessed. In the crush and end-to-end groups, soluble NRG1 mRNA increased early, with a peak at 1 day and a returning to baseline 3 days after injury. In denervated muscle this peak was not visible, and soluble NRG1 expression was not perturbed. Statistical analysis revealed differences between groups at 1 day. NRG1α and -β were differently modulated after nerve injury: the α isoform expression increased at day 1 in the crush and end-to-end repair groups and 14 days after injury in the crush and denervated groups; NRG1β was upregulated at 1 day after crush, whereas in the end-to-end group the upregulation was not statistically relevant with respect to un-injured muscle. Moreover, in the crush group the NRG1β expression returned to baseline at 28 days. In denervated muscle the expression of NRG1β did not change. Additionally, expression of the NRG1 type a, b, and c isoforms was differently modulated by nerve injury. The NRG1 type a expression pattern changed depending on the nerve injury: in the crush group it was strongly upregulated at 1 day and then returned to baseline; in the end-to-end repair group the expression increased and reached a peak at 28 days; and in the denervated group it was upregulated at 3 and 14 days. NRG1 type b was strongly upregulated 1 day after surgery in the crush and end-to-end repair groups. At 14 days an increase in NRG1 type b was detectable in the crush group and in the denervated group. In contrast, NRG1 type c showed downregulation at 1 day in the denervated muscle and at 3, 7 and 28 days in the crush group.

**Figure 3:**
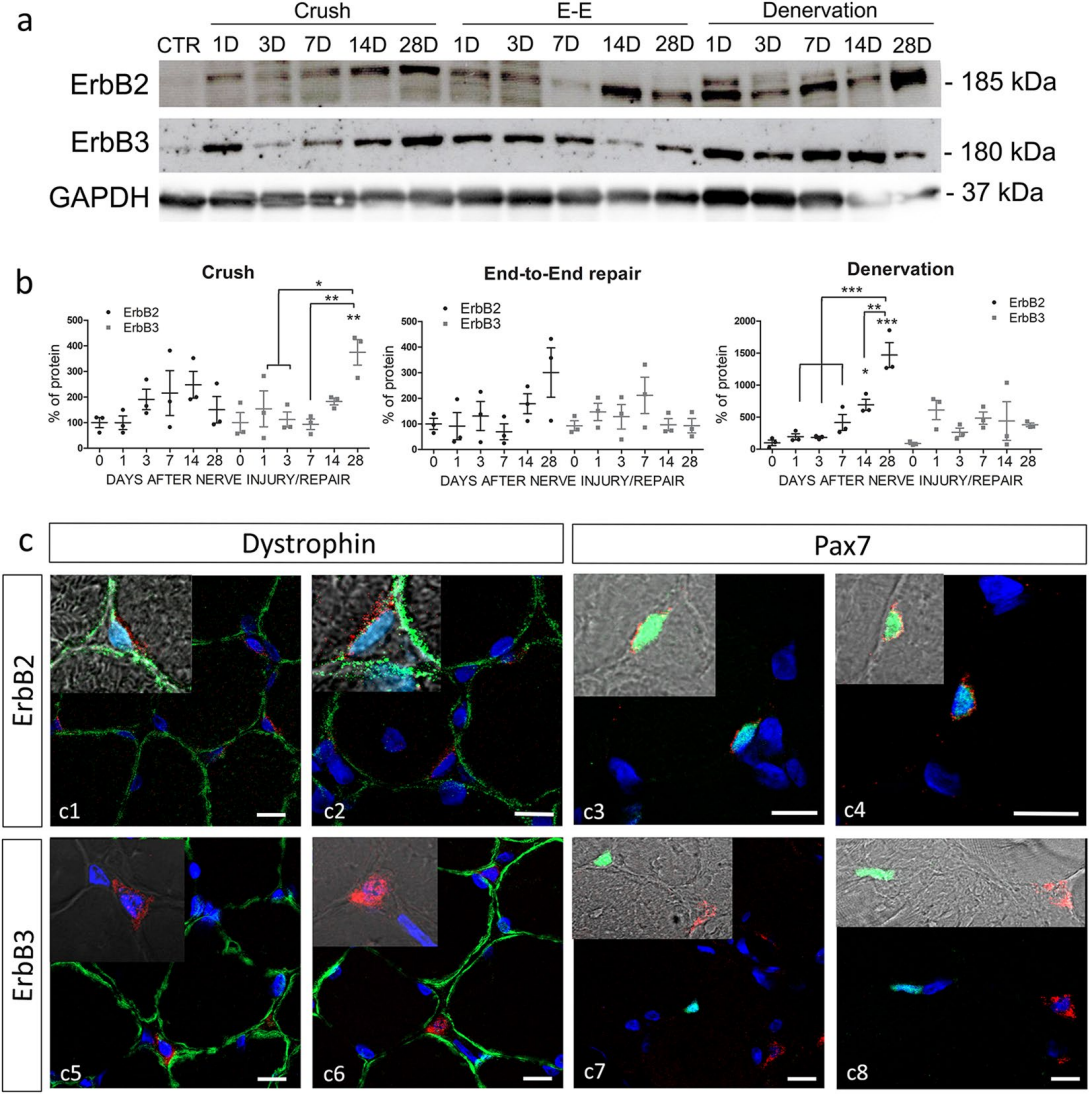
ErbB2 and ErbB3 protein expression in muscle after nerve injury. (**a**) Representative image of western blot analysis of ErbB2 and ErbB3 protein expression in SDF muscle after nerve injury. Three different types of nerve injury and injury/repair were performed on the median nerve (denervation, crush and end-to-end repair/E-E), and at different time points (D = days) muscle was collected and used for western blot analysis. CTR refers to un-injured muscle (control). (**b**) Graphs report the results of the quantitative analysis of ErbB2 and ErbB3 proteins in muscle samples. Three animals for each time-point were analysed for each group. Zero on x-bar refers to un-injured muscle. Statistical analysis: one-way ANOVA plus Bonferroni’s post hoc test: *p ≤ 0.05; **p ≤ 0.01; ***p ≤ 0.001. The significance versus un-injured muscle is indicated directly over the sample bar. (**c**) Representative images of immunofluorescence analysis performed on muscle tissue after 14 days crush nerve injury (c3,4,6,7,8) or muscle denervation (c1,2,5). Red staining: ErbB2 or ErbB3; green staining: dystrophin or Pax7; blue staining: DAPI. In the inserts, merge of DAPI, Dystrophin and ErbB2 (c1-c2), Pax7 and ErbB2 (c3-c4), DAPI and ErbB3 (c5-c6), Pax7 and ErbB3 (c7-c8) are shown. Scale bar: 10 µm.

The expression of ErbB2 and ErbB3 proteins in muscle tissue after nerve injury was analysed in biological triplicates corresponding to the samples analysed by qRT-PCR (Fig. 3a,b). After crush injury, ErbB2 protein level showed a non-significant increase with respect to un-injured muscle at 7 and 14 days, followed by a decrease at 28 days after nerve injury, while ErbB3 protein statistically increased at 28 days after injury. In the end-to-end repair group, ErbB2 protein showed a non-significant increase at day 28 (in accordance with the mRNA expression). ErbB3 protein seemed not to be regulated. Finally, after muscle denervation, ErbB2 protein was strongly upregulated, with peaks at 14 days and especially at 28 days after injury. ErbB3 protein seemed not to be regulated, even though its level was higher in injured than in un-injured muscle. Some discrepancies are visible between protein and mRNA regulation; however, we have to underline that protein analysis with western blot had a high animal-to-animal variability.

The localization of ErbB2 and ErbB3 in muscle tissue after denervation and nerve crush injury was investigated. The immunofluorescence analysis showed that ErbB2 is expressed in cells positive for Pax7 staining and closely associated with the sarcolemma, stained with Dystrophin (Fig. 3c). Thus, ErbB2 is expressed by satellite cells and, curiously, the expression of ErbB2 is concentrated in the membrane portion associated to the sarcolemma. Also ErbB3 protein was localized in cells outside to the muscle fibres, but these cells do not express Pax7 (Fig. 3c).

### Long-term analysis of muscle after denervation and reinnervation

**Figure 4:**
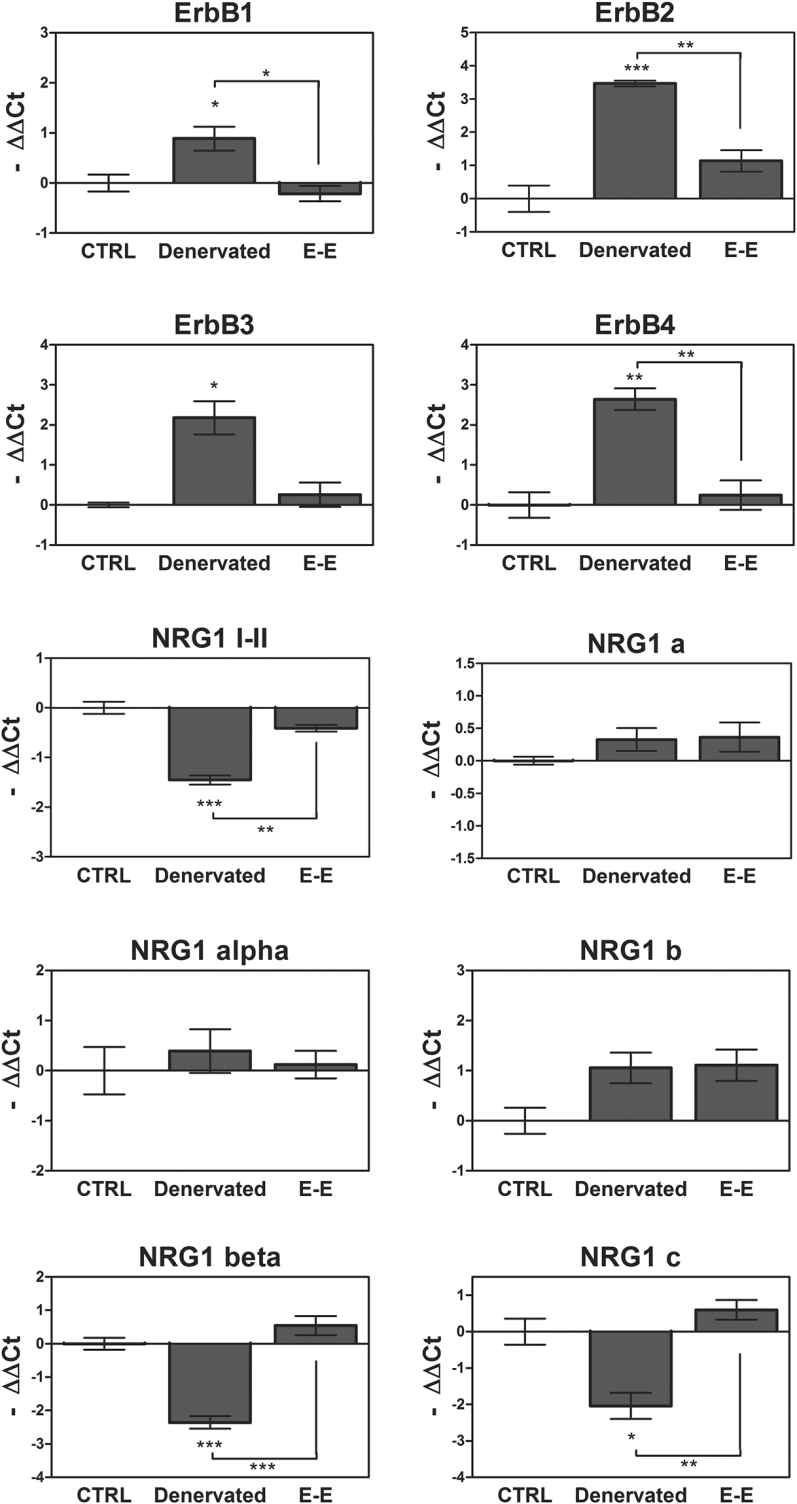
NRG1/ErbB expression after 12 weeks of muscle denervation and reinnervation. Twelve weeks after denervation or end-to-end repair (E-E), the expression of ErbB receptors and NRG1 was analysed by quantitative real-time PCR in SDF muscle. The analysis was performed on three animals per group; data are expressed as a mean ± SEM. The significant difference between the sample and the control is indicated over the sample bar. Statistical analysis: one-way ANOVA plus Bonferroni’s post hoc test: *p ≤ 0.05; **p ≤ 0.01; ***p ≤ 0.001.

We quantified the expression of ErbB receptors and NRG1 isoforms after long-term muscle denervation or end-to-end repair (12 weeks) (Fig. 4). All ErbB receptors were strongly upregulated in the denervated muscle, while in the end-to-end repair group their expression was similar to the control group. In the end-to-end repair group, the expression of soluble NRG1 (I-II) was comparable to the control muscle, whereas in the denervated muscle a decrease in its expression is detectable. Finally, the analysis of all NRG1 isoforms revealed that only NRG1β and NRG1 type c were highly downregulated after long-term denervation.

### *In vitro* analysis of NRG1 effects on atrophic myotubes

**Figure 5:**
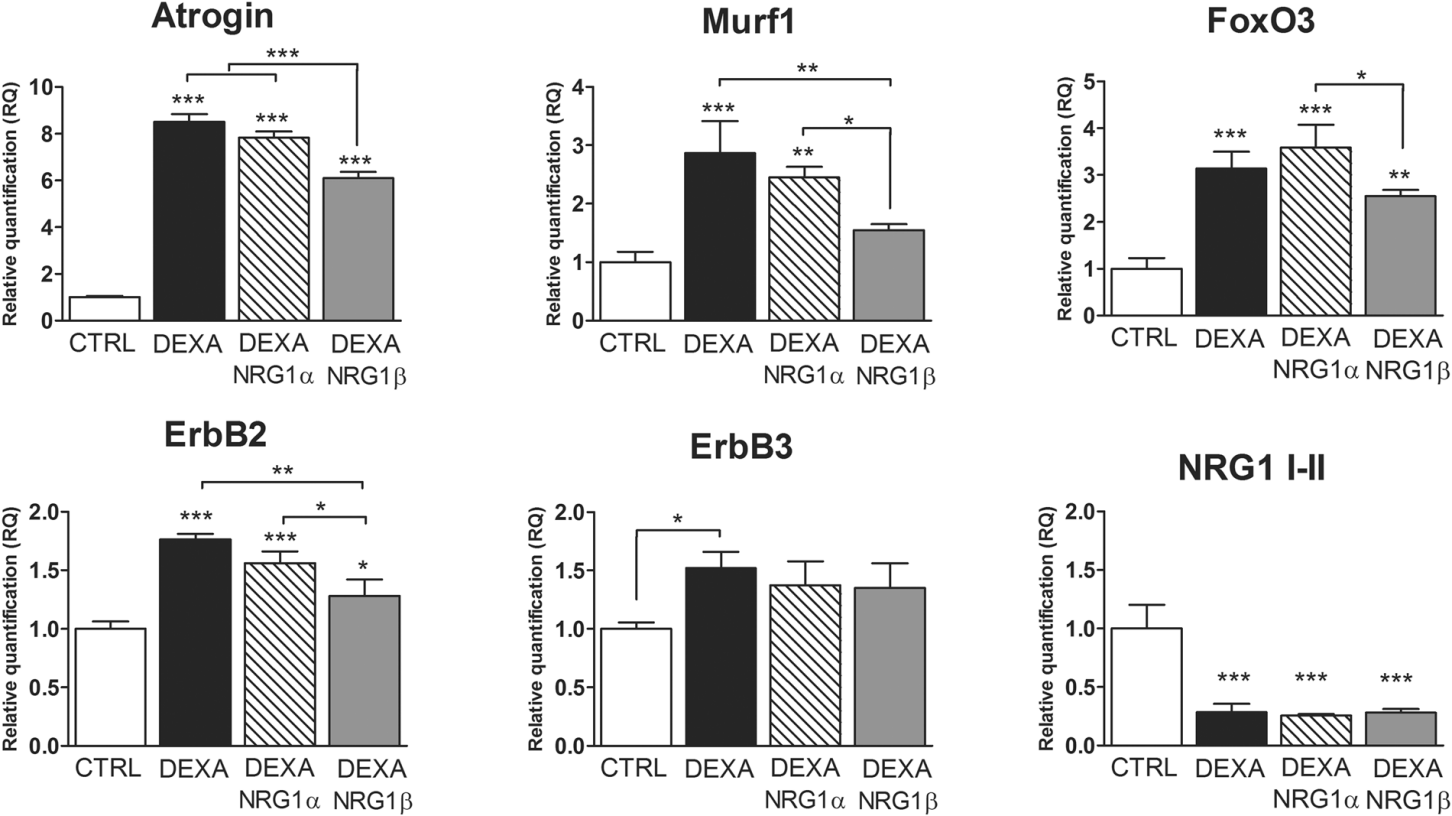
Dexamethasone-treated C2C12 myotubes as an *in vitro* model to study the NRG1/ErbB system during muscle atrophy. Twenty-four hours after 1 µM dexamethasone treatment, alone or in combination with 30 nM NRG1α or 5 nM NRG1β, C2C12 mRNA was extracted for quantitative real-time PCR analysis. Data refer to biological triplicates and are expressed as the mean ± SD. The significant difference between a sample and the control is indicated over the sample bar. Statistical analysis: one-way ANOVA plus Bonferroni’s post hoc test: *p ≤ 0.05; **p ≤ 0.01; ***p ≤ 0.001. DEXA: dexamethasone.

To study the NRG1/ErbB system during muscle atrophy and following treatment with NRG1, the C2C12 cell line was used as *in vitro* model of atrophic myotubes. In this simplified experimental model, the nervous component was missing and atrophy was pharmacologically induced by treatment with dexamethasone, a corticosteroid inducing an atrophic condition by upregulating two atrogenes, atrogin and murf1. The expression levels of these atrogenes were evaluated and, as expected, both were upregulated after dexamethasone treatment (Fig. 5). FoxO3 mRNA, a major atrogene activator, was also upregulated after treatment. C2C12 myoblasts and myotubes expressed ErbB1, ErbB2 and ErbB3, whereas ErbB4 mRNA was barely detectable. ErbB2 and ErbB3 expression in C2C12 myotubes increased in dexamethasone-induced atrophy, while a strong inhibition of soluble NRG1 I-II mRNA was observed (Fig. 5).

C2C12 myotubes were then treated with dexamethasone together with NRG1α or -β (Fig. 5). The results show that NRG1β induced a strong downregulation of murf1 mRNA, whose expression was similar to control, and small but significant reductions of atrogin and FoxO3 expression. In contrast, NRG1α treatment did not affect atrogin, murf1 or FoxO3 expression. Moreover, ErbB2 expression was decreased in the NRG1β group with respect to dexamethasone alone or NRG1α treatment. ErbB3 expression was not influenced by NRG1α or -β treatment. However, the expression of ErbB3 in C2C12 myotubes was already high and correlated with the differentiation stage. Neither NRG1 treatment affected soluble NRG1 expression.

**Figure 6:**
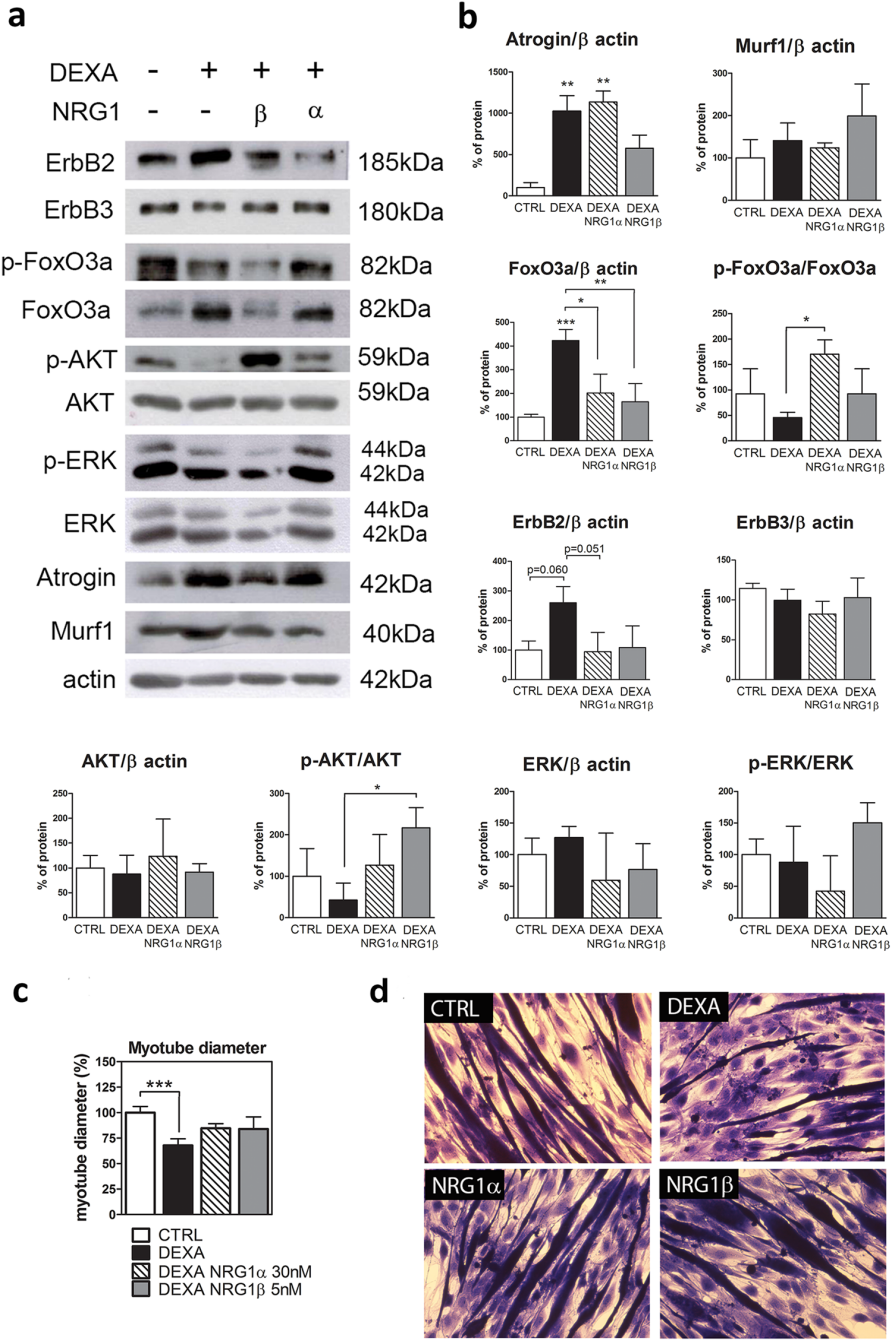
Molecular and morphological analysis of dexamethasone-induced atrophy in C2C12 myotubes treated with NRG1. (**a**) Representative pictures of western blot assays performed with proteins extracted from C2C12 myotubes treated for 24 h with 1 µM dexamethasone alone or in combination with 30 nM NRG1α or 5 nM NRG1β. (**b**) C2C12 myotube protein quantification. (**c**) Myotube diameter measurement. C2C12 cells were treated for 24 h with dexamethasone and then for 48 h with dexamethasone alone or in combination with NRG1α or NRG1β. The significant difference between a sample and the control is indicated over the sample bar. (**d**) Representative images used for myotube diameter measurement. Data refer to a biological triplicate and are expressed as the mean ± SD. Statistical analysis: one-way ANOVA plus Bonferroni’s post hoc test: *p ≤ 0.05; **p ≤ 0.01; ***p ≤ 0.001. DEXA: dexamethasone.

Protein analysis confirmed the presence of higher levels of atrogin and FoxO3 protein in the atrophic condition (Fig. 6a,b). Treatment with NRG1β reduced both atrogin and FoxO3 levels, while NRG1α downregulated only FoxO3 protein. The phosphorylation of FoxO3, corresponding to the inactivation of the protein, was significantly higher in NRG1α treatment compared to dexamethasone alone. Surprisingly, murf1 protein was unchanged under all conditions. The administration of NRG1, either -α or -β, rescued the ErbB2 level (Fig. 6a,b). ErbB3 protein did not change under any conditions. Then, we analysed the two main signalling pathways activated by ErbB2/ErbB3: the PI3-kinase pathway and the MAPK pathway, evaluating the phosphorylation of AKT and ERK. AKT phosphorylation was strongly induced by NRG1β treatment, while ERK phosphorylation did not change significantly between groups (Fig. 6a,b).

Additionally, we analysed the mean myotube diameter after NRG1 treatment, as an atrophic index. After dexamethasone treatment, myotube diameter was lower than control, as expected; this difference was lost in the NRG1α and -β groups, even if the rescue was not complete (Fig. 6c,d).

## Discussion

The aim of this study was to describe the modulation of the NRG1/ErbB system in skeletal muscle after peripheral nerve injury. To investigate the molecular changes occurring in muscle during denervation and reinnervation, we took advantage of three nerve injury models characterized by different severities, namely denervation (chronic injury), crush injury (acute injury followed by fast recovery) and end-to-end repair (acute injury followed by slow recovery).

We analysed muscle morphology after the most severe nerve injury (the irreversible denervation) and after the faster reinnervation model (crush injury) to follow histological changes in these two opposite conditions. In both models, nerve fibres undergo Wallerian degeneration early after nerve injury. In denervation, nerve fibres degenerate over time^17^, while after crush injury a regeneration phase is observed already at 14 days, and mature nerve fibres are present at 28 days. So crush injury represents a good model to observe the effects of denervation and reinnervation processes in a short time window: 12 days after median nerve injury, a functional recovery of muscle is already detectable, and a 75% rescue of muscle activity is reached after 28 days^18^. Accordingly, in this study we found that, after denervation, the muscle atrophy worsened over time, while the muscle mass reduction detectable at 7 days after crush injury was restored at 14 and 28 days. The muscle fibre size distribution confirmed these data: 28 days after crush injury, the distribution was similar to the un-injured muscle; in contrast, in the denervated muscle the number of smaller fibres was higher, consistent with literature data^19^.

NRG1 and ErbB receptors are well-known regulators of nerve growth and maintenance. Their involvement in the peripheral nerve regeneration process is well studied^17,20,21^. However, little is known about the modulation of NRG1 and ErbB receptors in muscle tissue after nerve injury. Since in skeletal muscle NRG1 regulates neuromuscular junction gene expression, acetylcholine receptor numbers, muscle homeostasis, and satellite cell survival^6,15,22^, we can postulate that alterations of nerve–muscle crosstalk also modify the NRG1/ErbB system. In our previous work and in other studies, the expression of NRG1 was analysed in the denervated muscle using RT-PCR, but the results were partially discordant, while ErbB2, ErbB3 and ErbB4 receptors were upregulated^11,23,24^. Here we extended the expression analysis to different isoforms of NRG1 and ErbB receptors in three models of nerve injury to differentiate between muscle denervation and slow and fast muscle reinnervation, using quantitative real-time PCR for a time-course analysis.

In coherence with our previous observation, we found that ErbB2 and ErbB3 receptors were upregulated in denervated muscle over time (indeed, at 12 weeks they are still upregulated). In contrast, ErbB2 and ErbB3 expression in the reinnervated muscle seemed to be inversely correlated with muscle functional recovery. In the crush group their expression returned to baseline 28 days after injury, whereas in the end-to-end repair group their level decreased later, at 12 weeks, in accordance with the muscle functional recovery as assessed by the grasping test^18^. Also ErbB4 was upregulated in denervated muscle over time, while in both reinnervated muscle groups it returned to baseline 28 days after injury, indicating that this receptor might be mainly involved in the early phases of muscle reinnervation. Because ErbB4 is specifically localized at the neuromuscular junction^25^, we can speculate that its return to basal expression level might reflect nerve–muscle reconnection.

Interestingly, ErbB2 and ErbB3 protein expression did not always correlate with the mRNA level, suggesting that post-transcriptional regulation affects ErbB translation and protein stability. In fact, on one hand, we observed an upregulation of ErbB2 protein at 14 and 28 days after denervation, in line with the expression analysis; on the other hand, no changes in ErbB2 protein expression were detected after crush injury or after end-to-end repair, nor in ErbB3 in the end-to-end repair group. Curiously, we found high level of ErbB3 protein at 28 days after nerve crush injury; this upregulation was shifted in time with respect to mRNA expression, whose peak was at 14 days. In denervated muscle, ErbB3 protein showed an increasing trend with respect to un-injured muscle; however, this trend was not statistically significant, but we have to underline the high animal-to-animal variability observed in western blot analysis. The upregulation of ErbB receptors might be correlated to satellite cell activation. Quiescent satellite cells do not express ErbB receptors, but after activation *in vitro* they express all ErbB receptors^25^. At 14 and 28 days after nerve injury, we found that both ErbB2 and ErbB3 proteins were localized in cells associated to the plasmalemma, but only ErbB2 expression is clearly ascribable to satellite cells (Pax7 positive cells). The role of ErbB3 protein in controlling muscle myogenesis and the maintenance of muscle precursor is still obscure. Nevertheless, increased ErbB3 expression has been observed in C2C12 and L6 cell lines after myotube differentiation, correlated with early upregulation of NRG1α^8,26^. The high level of ErbB2 and ErbB3 in the denervation phase might be necessary to the response to NRG1α which, in L6 cells, stimulates cell fusion and myotube formation in a dose-dependent manner^26^.

As far as we know, this is the first study showing modulation of several NRG1 isoforms (not only α and β but also type a, b and c) in skeletal muscle after nerve injury. We confirmed the upregulation of NRG1α 14 days after denervation^23^. Intriguingly, 1 day after nerve injury, NRG1α and -β were differentially modulated according to the injury severity, suggesting that their expression might be affected by the integrity of the nerve: where only axon continuity is lost (crush injury), both NRG1α and -β are upregulated; where nerve injury is more severe and nerve continuity is lost, but immediately repaired (end-to-end repair), only NRG1α is upregulated; finally, where nerve injury is most severe and nerve continuity is completely lost (denervation), both NRG1 isoforms are not upregulated. After toxin-induced injury, NRG1 is upregulated in muscle during regeneration, although it is not known whether this was the α or β isoform(s)^16^. Taken together, these data indicate that nerve signals might promote muscle regeneration after injury influencing NRG1 expression and that NRG1α might be the principal player in the muscle response to nerve injury. In the past, the difference between NRG1α and -β was thought to be only quantitative, with α isoforms 10-fold less active than β isoforms; on the contrary, the activity of NRG1α and β can be qualitatively different^27,28^.

We also analysed NRG1 types a, b and c, which differ in their intracellular domains and have never been studied in skeletal muscle. We observed a similar expression pattern for NRG1 types a and b, showing different expression peaks in all the experimental models, while NRG1 type c was downregulated. These data suggest that NRG1 type a and b might be involved in the response to the injury, as previously observed in nerve tissue^17^. Although the role of these NRG1 isoforms is poorly studied, it is known that the NRG1 intracellular C terminus domain is involved in back-signalling: it can be cleaved and transported to the nucleus, where it can modulate gene expression. In neurons, for example, it represses several apoptosis regulators^29^. However, further studies are needed to understand the roles of these isoforms in denervated and reinnervated muscle.

To explain the different roles of NRG1 isoforms observed in denervated and reinnervated muscle *in vivo*, we investigated the effect of NRG1 in an *in vitro* atrophy model, represented by C2C12 cells treated with dexamethasone. Despite the limits associated with *in vitro* models, myoblast/myotube models are widely used and represent a useful tool to study muscle atrophy mechanisms^30,31^. Our model is far from mimicking denervation-induced atrophy, being devoid of the neuronal component. Thus, the modulation of the NRG1/ErbB system was analysed in this model of pharmacological-induced atrophy. The analysis confirmed the upregulation of ErbB2/ErbB3 mRNA and ErbB2 protein in correlation with the upregulation of atrophic markers after dexamethasone treatment, suggesting that ErbB upregulation can be associated with the atrophic condition triggered by different causes. Since NRG1α and NRG1β are upregulated early after denervation/reinnervation and downregulated at early and late stages of complete denervation *in vivo*, we hypothesized that exogenous administration of NRG1 could rescue the atrophic condition. In the muscle, the action of NRG1β depends on the differentiation stage of muscle cells: it induces myogenesis and blocks differentiation in myoblasts^9^, while it stimulates differentiation in myotube cultures^32^. Moreover, in the L6 myoblast cell line, NRG1α stimulates cell fusion and myotube formation^26^. Here, we demonstrated that both proteins had an effect on atrophic C2C12 in terms of myotube diameter, although only NRG1β reduced atrogin, murf1 and FoxO3 expression levels. Interestingly, NRG1α induced FoxO3 phosphorylation and reduced FoxO3 and ErbB2 protein levels. We speculate that NRG1α effect is delayed in time with respect to NRG1β, as both restore myotube diameter over a longer time frame, supporting the thesis of quantitative differences between the two isoforms. Nevertheless, we must keep in mind that only myotubes and not myofibres were present in our *in vitro* culture, thus limiting the effect (or the detection of the effect) exerted by NRG1. We cannot exclude that NRG1 has additional effects that altered our analysis. In fact, though most of the cells in culture were differentiated, there was a small component of myoblast cells which might have responded to NRG1 treatment, altering for example the fusion rate.

In summary, our results demonstrate that the regulation of the NRG1/ErbB system in muscle after nerve injury correlates with the denervation and reinnervation phases. The different expression patterns observed for the NRG1 isoforms suggest a specific role for each isoform. Our data highlight the importance of studying the NRG1/ErbB system not only in a peripheral nerve perspective but also in a target muscle perspective. Our experiments demonstrate active roles of NRG1α and -β to rescue atrophic conditions induced by dexamethasone *in vitro*. Treating muscle with NRG1 after nerve injury is promising, especially in light of the recent findings that NRG1 injection in muscle increases the size of the AChR cluster at NMJ^33,34^ and that NRG1 over-expression in muscle accelerates the collateral sprouting of regenerating nerves^35^. Future experiments will be needed to address the *in vivo* efficacy of NRG1, and the challenge will be to identify the roles exerted by the different NRG1 isoforms.

## Material and Methods

### Animals

All procedures were approved by the Bioethics Committee of the University of Torino, by the Institutional Animal Care and Use Committee of the University of Torino, and by the Italian Ministry of Health, in accordance with the European Communities Council Directive European Communities Council (2010/63/EU), the National Institutes of Health guidelines and the Italian Law for Care and Use of Experimental Animals (DL26/14).

### Surgery

A total of 73 adult female Wistar rats (Harlan Laboratories), weighing approximately 200 g, were used. Animals were housed at a constant temperature and humidity, under 12–12 h light/dark cycles, with free access to food and water. Animals were randomly divided into four experimental groups: (i) crush group (axonotmesis): the median nerve was crushed at the mid-humerus level with a non-serrated clamp, as previously described^18^; (ii) end-to-end repair (neurotmesis): the median nerve was transected and immediately repaired with direct suture; (iii) denervated muscle group: the median nerve was transected, and the proximal stump was sutured to the major pectoralis muscle to avoid regeneration, while the distal nerve stump was allowed to degenerate over time; (iv) control group: the median nerve was not injured. Rat were sacrificed at different time points until 28 days after the surgery. For the molecular studies, six animals were sacrificed at a longer time point (12 weeks, n = 3) in the end-to-end and denervated groups.

### Morphological and morphometrical analysis

Morphological analysis was performed after median nerve crush or denervation. SDF muscles were harvested 7, 14 and 28 days after nerve injury and were immediately embedded in OCT, frozen in cold isopentane (−50 °C) and then stored at −80 °C. All samples were cut with a cryostat to obtain transverse sections (10 μm thickness), then stored at −20 °C. Haematoxylin-eosin staining was then performed, after a quick passage in 70% ethanol to fix and lay the section on glass slide. Morphological analysis was performed using a DM4000B microscope and DFC320 digital camera, using the IM50 image manager system (Leica Microsystems). Samples were processed as follows: adjacent non-overlapped fields were acquired at 10 × magnification to cover all section areas, and these images were used to evaluate fibre diameter. We used the systematic protocol previously described for nerve tissue^36^. For each group, at least four animals were analysed; for each muscle, one section was analysed.

### RNA isolation, cDNA preparation and quantitative real-time PCR (qRT-PCR) analysis

After mechanical dissociation of frozen muscle, tissue was dissolved in TRIzol (Thermo Fisher Scientific) and processed according to the manufacturer’s instructions. Three animals for each time point were analysed. For each sample, 1 μg total RNA was reverse-transcribed (RT)^16^. The obtained cDNA was diluted 1:10 with water and stored at −20 °C.

Quantitative real-time PCR analysis was performed using an ABI Prism 7300 (Applied Biosystems). The reaction was performed in at a volume of 25 µl containing 5 µl of diluted cDNA (corresponding to 25 ng of starting RNA), 1 × Sybr Green PCR Master Mix (BioRad) and 300 nM forward and reverse primers. All primers were manually designed to amplify specific isoforms of NRG1 or ErbB receptors (Tables 1 and 2). The dissociation curve was analysed to check the quality of the reaction. Data were analysed by the ΔΔCt relative quantification method, normalizing to the housekeeping gene TATA-box binding protein (TBP). We determined the difference between Ct values of target and housekeeping gene (ΔCt), the difference between the ΔCt values of the samples and the ΔCt mean value of control sample was then calculated (ΔΔCt). Data about the time-course analysis on muscle tissues are reported as −ΔΔCt instead of 2^−ΔΔCt^ (RQ) because this way is more suitable to appreciate both up- and downregulation.

**Table 1:** Primer used for qRT-PCR analysis in rat skeletal muscle.

GENE	ACCESSION NUMBER	PRIMER FORWARD	PRIMER REVERSE	AMPLICON SIZE
ErbB1	NM_031507	5′-CACCACGTACCAGATGGATG-3′	5′-CGTAGTTTCTGGGGCATTTC-3′	83
ErbB2	NM_017003	5′-TGACAAGCGCTGTCTGCCG-3′	5′-CTTGTAGTGGGCGCAGGCTG-3′	106
ErbB3	NM_017218.2	5′-CGAGATGGGCAACTCTCAGGC-3′	5′-AGGTTACCCATGACCACCTCACAC-3′	129
ErbB4	AY375307.1	5′-AAGTTCTGGATGCGGAAGATGCC-3′	5′-TTGTTCAGCACACACAGTCCTGG-3′	103
NRG1 type I/II	AF194993	5′-GGCGCAAACACTTCTTCATCCAC-3′	5′-AAGTTTTCTCCTTCTCCGCGCAC-3′	82
NRG1 alpha	AF194439	5′-TGCGGAGAAGGAGAAAACTTTC-3′	5′-TTGCTCCAGTGAATCCAGGTTG-3′	113
NRG1 beta	AF194438	5′-TGCGGAGAAGGAGAAAACTTTC-3′	5′-AACGATCACCAGTAAACTCATTTGG-3′	119
NRG1 type a	U02322	5′-CCCCTGACTCCTACAGAGACTCTCC-3′	5′-TAGGGGAGCTTGGCGTGTGG-3′	107
NRG1 type b	AY973245	5′-CCCCTGACTCCTACAGAGACTCTCC-3′	5′-AGGGTCTAAGATGAGTTGCTGACAGC-3′	126
NRG1 type c	U02324	5′-CCCCTGACTCCTACAGAGACTCTCC-3′	5′-CCGCAGGTGCTCATGGGATTC-3′	108
TBP	NM_001004198.1	5′-TAAGGCTGGAAGGCCTTGTG-3′	5′-TCCAGGAAATAATTCTGGCTCATAG-3′	68

**Table 2:** Primer used for qRT-PCR analysis in C2C12 mouse cell line.

GENE	ACCESSION NUMBER	PRIMER FORWARD	PRIMER REVERSE	AMPLICON SIZE
Atrogin	NM_026346	5′-TCACAGCTCACATCCCTGAG-3′	5′-GTGTAGAGAGTCTGGAGAAGTTCCCG-3′	131
ErbB1	NM_207655	5′-GCTGGTGTTGCTGACCGCG-3′	5′-GGGTGAGCCTGTTACTTGTGCC-3′	86
ErbB2	NM_001003817	5′-GCAAGCACTGTCTGCCATGC-3′	5′-GGGCACAAGCCTCACACTGG-3′	96
ErbB3	NM_010153	5′-CGAGATGGGCAACTCTCAGGC-3′	5′-AGGTTACCCATGACCACCTCACAC-3′	129
ErbB4	NM_010154	5′-CATGGCCTTCCAACATGACTCTGG-3′	5′-GGCAGTGATTTTCTGTGGGTCCC-3′	108
FoxO3	NM_019740	5′-GCGCTGTGTGCCCTACTTCAAG-3′	5′-CTCTTTCCCCCATCGGGGTTG-3′	165
Murf1	NM_001039048	5′-TACCAAGCCTGTGGTCATCC-3′	5′-AACGACCTCCAGACATGGAC-3′	128
NRG1 type I/II	AY648976.1	5′-CATGTCAGCCTCAACTGAAAGACCC-3′	5′-TGGTCCCAGTCGTGGATGTAGATG-3′	116
NRG1 alpha	NT_039457	5′-TGCGGAGAAGGAGAAAACTTTC-3′	5′-TTGCTCCAGTGAATCCAGGTTG-3′	114
NRG1 beta	NM_178591	5′-GTGCGGAGAAGGAGAAAACTTTC-3′	5′-AACGATCACCAGTAAACTCATTTGG-3′	117
TBP	NM_013684.3	5′-GATCAAACCCAGAATTGTTCTCC-3′	5′-GGGGTAGATGTTTTCAAATGCTTC-3′	106

### Immunofluorescence analysis

Transverse muscle sections were fixed with 4% PFA for 5 min at room temperature and washed twice with PBS. The permeabilization was performed in methanol for 3 min at −20 °C. Slices were then washed twice in a solution of PBS and 0.1 M glycine for 5 min and incubated for 40 min at room temperature in a solution of PBS, 2% goat serum (DAKO) and 1% BSA. The primary antibodies were incubated overnight in PBS with 1% BSA: anti-ErbB2 (1:200, #2165, Cell Signaling); anti-ErbB3 (1:200, #12708, Cell Signaling), anti-dystrophin (1:400, #D8168, Sigma). For Pax 7 (1:10, purchased by Hybridoma Bank) staining an additional step was performed after the permeabilization: slides were incubated 7 minutes at 500 W in microwave in a hot antigen retrieval solution (10 mM citrate buffer pH 6, obtained from 0.1 M citric acid and 0.1 M sodium citrate) and then washed three times with PBS. The secondary antibodies used were goat-anti-mouse Alexa Fluor 488 (1:200, Invitrogen) and goat-anti-rabbit Cy3 (1:400, Jackson). Nuclei were stained with DAPI (1:1000, Sigma).

### C2C12 cell culture

C2C12 are a mouse cell line (ATCC^®^ CRL-1772^™^) derived from a subclone of myoblasts. Cells were grown in Dulbecco’s Modified Eagle’s Medium, (#30–2002, DMEM, Invitrogen) supplemented with 100 U/mL penicillin, 100 *μ*g/mL streptomycin, 1 mM sodium pyruvate, 2 mM l-glutamine and 10% heat-inactivated foetal bovine serum (Invitrogen). Cells were grown until 80% confluence, and then the medium was changed to differentiation medium (DMEM supplemented with 100 U/mL penicillin, 100 *μ*g/mL streptomycin, 1 mM sodium pyruvate, 2 mM l-glutamine and 2% horse serum). The experiments were performed after five days of differentiation. For the molecular analysis, differentiated cells were treated as follows: 1 µM dexamethasone (D2915, Sigma) was added to the differentiation medium, alone or in combination with recombinant peptides corresponding to the EGF-like domain of NRG1α (30 nM; #296-HR) or NRG1β (5 nM, #396-HB) purchased from R&D systems, for 24 h. For myotube diameter analysis, cells were treated for 24 h with 1 µM dexamethasone, followed by 48 h treatment with dexamethasone alone or in combination with NRG1α or NRG1β. Cells were then fixed in 2% glutaraldehyde and stained with 1% toluidine blue. Five random pictures were taken for each well using a Nikon eclipse TS100 microscope. Mean myotube diameter was calculated using ImageJ (National Institutes of Health, USA). At least 40 diameters were manually counted for each well, collecting three measurements for each myotube.

### Total protein extraction and western blot analysis

Proteins were extracted using boiling Laemmli buffer (2.5% SDS, 0.125 M Tris–HCl pH 6.8). Frozen pieces of muscle or C2C12 cells were mechanically dissociated in Laemmli buffer and incubated at 100 °C for 3 min. Primary antibodies used were anti-ErbB2 (1:1000, #sc-284), anti-ErbB3 (1:1000, #sc-285), anti-β-actin (1:4000, #sc-2228), anti-Murf1 (1:4000, #sc-32920) (Santa Cruz); anti-p-FoxO3a (Thr32) (1:1000, #9464), anti-FoxO3a (1:1000, #2497), anti-AKT (1:1000, #9272), anti-p-AKT (1:1000, #4051), anti-ERK (1:1000, #9102), anti-p-ERK (1:1000, #9106) (Cell Signalling); anti-Atrogin (1:3000, #ab168372, Abcam). The secondary antibodies used were ECL^TM^ anti-rabbit IgG (1:40000, #NA934) and ECL^TM^ anti-mouse IgG (1:40000, #NA931) (GE Healthcare). All the antibodies used detected a band of the predicted molecular weight. ImageJ was used for a quantitative analysis of the western blot bands obtained.

### Statistical methods

The statistical analysis was performed using SPSS Statistics 22.0 software (IBM, USA). Gene expression analysis for muscle tissue was performed in biological and technical triplicate. Data are reported as the mean ± SEM, and one-way ANOVA followed by Bonferroni’s post hoc test was used. We carried out two analyses: the first considering each single experimental group at different time points (Table S1), the second considering single time points and comparing the three experimental groups (only this analysis is reported in figures). Protein analysis was performed on muscle samples from three animals for each analysed time point. Data are reported as the mean ± SEM, and one-way ANOVA followed by Bonferroni’s post hoc test was used for the statistical analysis. Cell experiments were performed in biological triplicates (three different cell differentiation experiments were used for a single analysis); all data are presented as the mean ± SD. One-way ANOVA followed by Bonferroni’s post hoc test was used to assess statistical significance. To compare two experimental groups directly, Student’s T-test was used. Differences between conditions were considered statistically significant at *P* ≤ 0.05.

### Data availability

All data generated or analyzed during this study are included in this article.

## Electronic supplementary material

Supplementary information
