# Author Correction: Modulation of the Neuregulin 1/ErbB system after skeletal muscle denervation and reinnervation

**DOI:** 10.1038/s41598-020-68521-1

**Published:** 2020-07-10

**Authors:** Michela Morano, Giulia Ronchi, Valentina Nicolò, Benedetta Elena Fornasari, Alessandro Crosio, Isabelle Perroteau, Stefano Geuna, Giovanna Gambarotta, Stefania Raimondo

**Affiliations:** 1 Department of Clinical and Biological Sciences, University of Torino, 10043 Orbassano, Italy; 2 Neuroscience Institute Cavalieri Ottolenghi (NICO), University of Torino, 10043 Orbassano, Italy; 3 Microsurgery Unit, AOU Città Della Salute E Della Scienza, PO CTO, 10126 Torino, Italy

Correction to: *Scientific Reports* 10.1038/s41598-018-23454-8, published online 22 March 2018

This Article contains an error. In Figure 1B, the panel for 14 days denervation is incorrect. The correct Figure 1 appears below.

**Figure 1:**
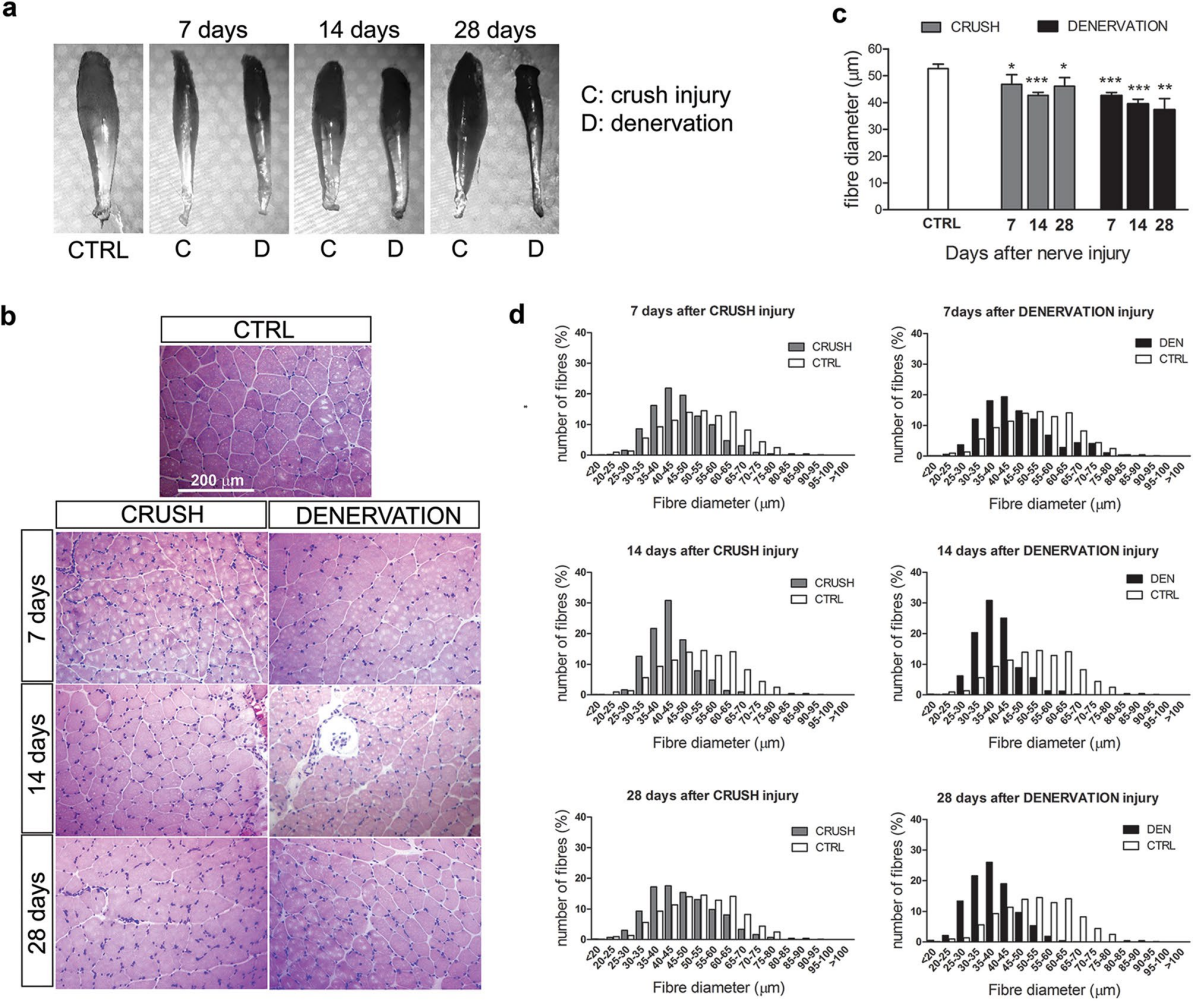
.

