# Supplementary material for: Modulation of the Neuregulin 1/ErbB system after skeletal muscle denervation and reinnervation
Source: Sci Rep. 2018 Mar 22;8:5047. doi: 10.1038/s41598-018-23454-8 (PMC5864756; doi:10.1038/s41598-018-23454-8)
Supplement: Supplementary file 1 — Supplementary information [file 41598_2018_23454_MOESM1_ESM.pdf]

Modulation of the Neuregulin1/ErbB system after skeletal muscle denervation and reinnervation

Michela Morano[1,2], Giulia Ronchi[1,2], Valentina Nicolò[1], Benedetta Elena Fornasari[1], Alessandro Crosio[3], Isabelle Perroteau[1], Stefano Geuna[1,2], Giovanna Gambarotta*[1], Stefania Raimondo[1,2].

[1] Department of Clinical and Biological Sciences, University of Torino, 10043 Orbassano, Italy
[2] Neuroscience Institute Cavalieri Ottolenghi (NICO), University of Torino, 10043 Orbassano, Italy
[3] Microsurgery Unit, AOU Città della Salute e della Scienza, PO CTO, 10126 Torino, Italy

* Corresponding author: giovanna.gambarotta@unito.it

SUPPLEMENTARY INFORMATION

Supplementary Table S1. Statistical analysis of real-time data for SDF muscle

type of injury	CRUSH		
**gene**	**time point**	**fold change**	**p- value**
ErbB2	7 days	3.28 ± 0.73	0.011
ErbB3	3 days	2.67 ± 0.83	0.031
	14 days	2.71 ± 0.09	0.022
ErbB4	7 days	2.57 ± 0.8	0.047
	14 days	3.32 ± 0.39	0.006
NRG1 I-II	1 day	8.35 ± 3.95	0.002
NRG1 α	1 day	4.66 ± 1.65	0.001
	14 days	3.26 ± 0.97	0.015
NRG1 β	1 day	12.44 ± 3.34	0.045
NRG1 type a	1 day	13.7 ± 12.5	0.014
NRG1 type b	1 day	13.9 ± 7.85	0.005
	14 days	10.7 ± 6.37	0.011
NRG1 type c	3 days	-1.44 ± 0.58	0.012
	7 days	-5.64 ± 0.13	0.000
	28 days	-2.87 ± 1.6	0.014
**type of injury**	**END-TO-END REPAIR**		
**gene**	**time point**	**fold change**	**p- value**
ErbB2	14 days	7.92 ± 3.33	0.007
	28 days	6.88 ± 3.0	0.006
ErbB3	3 days	3.75 ± 1.37	0.013
	28 days	2.81 ± 0.39	0.026
NRG1 I-II	1 day	5.08± 2.78	0.05
NRG1 α	1 day	6.87 ± 2.26	0.085
NRG1 type a	28 days	3.44 ± 1.17	0.036
NRG1 type b	1 day	5.30 ± 1.8	0.05
**type of injury**	**DENERVATED**		
**gene**	**time point**	**fold change**	**p- value**
ErbB2	7 days	4.11 ± 1.62	0.003
	28 days	9.95 ± 2.25	0.000
ErbB3	3 days	2.45 ± 0.1	0.008
	7 days	2.14 ± 0.37	0.029
	28 days	2.92 ± 0.75	0.002
NRG1 α	14 days	4.14± 2.2	0.023
NRG1 type a	3 days	2.73± 0.35	0.039
	14 days	10.08 ± 4.15	0.000
NRG1 type b	14 days	12.12 ± 2.75	0.007
NRG1 type c	1 day	-3.03 ± 1.02	0.004
	12 weeks	0.25 ± 0.08	0.027
NRG1 I-II	12 weeks	0.37 ± 0.04	0.000
NRG1 β	12 weeks	0.11 ± 0.03	0.01

Supplementary Figure S1

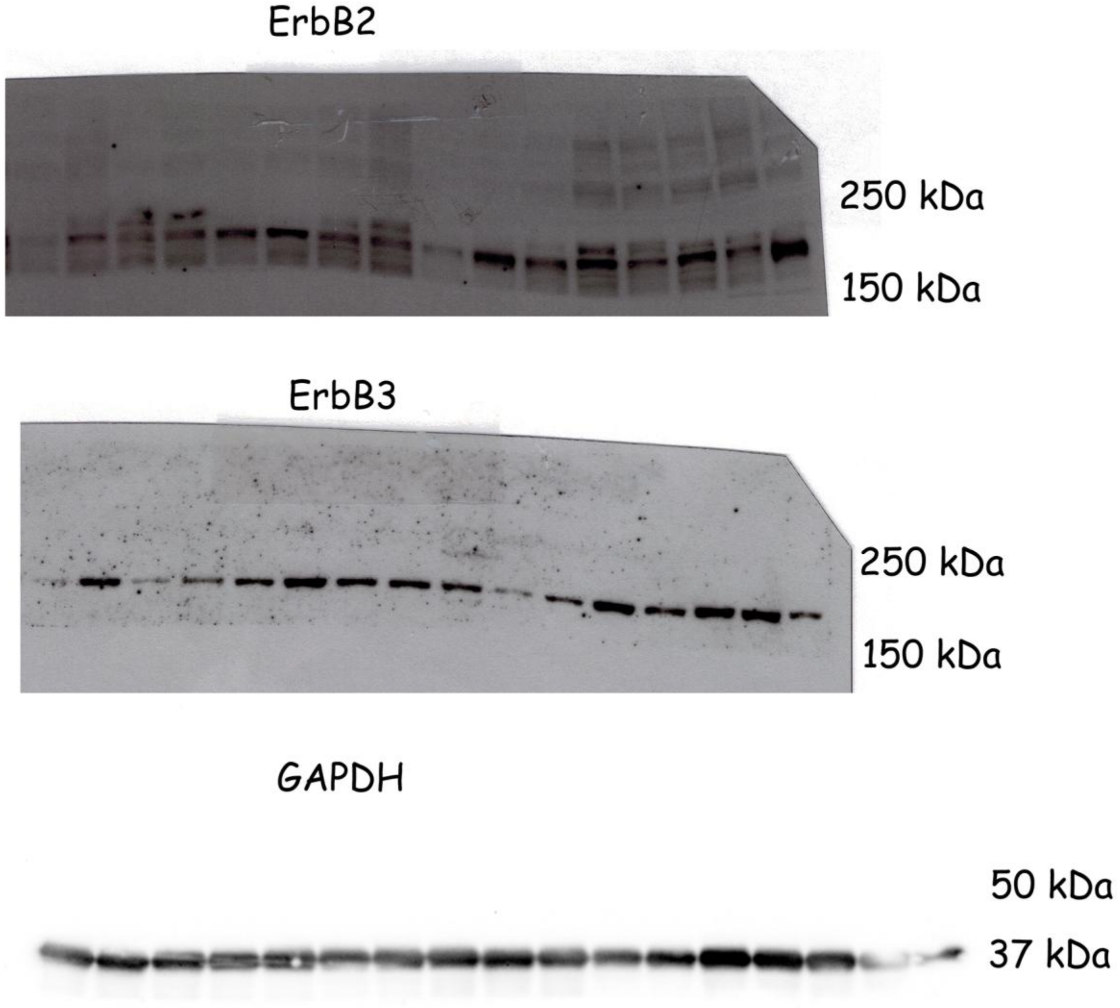

Figure S1. Full length western blots. Full length western blot for figure 3. The GAPDH band image was acquired using Chemidoc XRS (Biorad) and Quantity One software.

Supplementary Figure S2

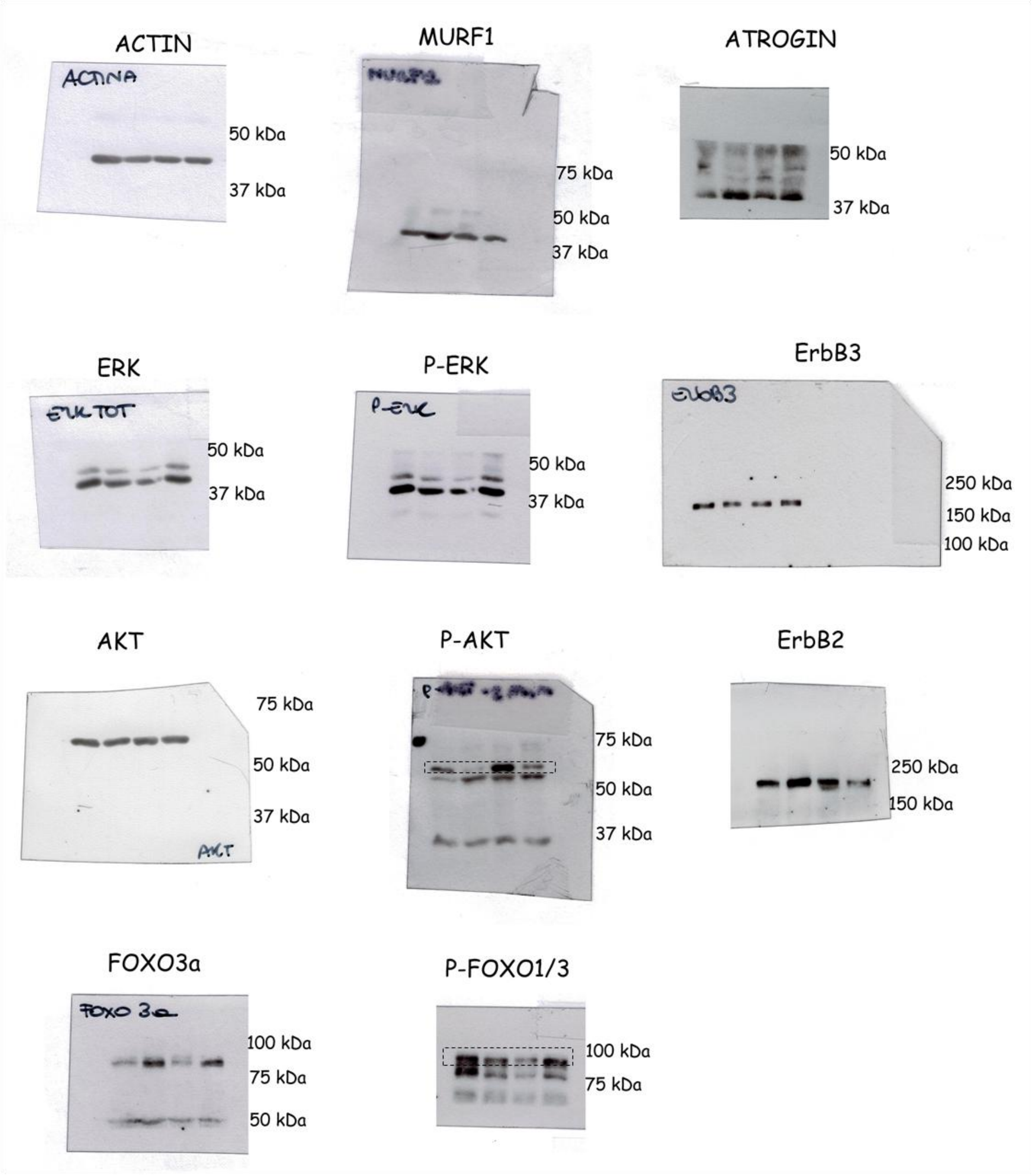

Figure S2. Full length western blots. Full length western blot for figure 6. The dotted box region indicates, when there is more than one visible band, the cropped portion of the blot shown in the main figure.
